# Early-Life Stressors, Personality Development, and Fast Life Strategies: An Evolutionary Perspective on Malevolent Personality Features

**DOI:** 10.3389/fpsyg.2018.00305

**Published:** 2018-03-12

**Authors:** Árpád Csathó, Béla Birkás

**Affiliations:** Medical School, Institute of Behavioral Sciences, University of Pécs, Pécs, Hungary

**Keywords:** life history theory, early-life stress, fast life strategies, personality development, the Dark Triad

## Abstract

Life history theory posits that behavioral adaptation to various environmental (ecological and/or social) conditions encountered during childhood is regulated by a wide variety of different traits resulting in various behavioral strategies. Unpredictable and harsh conditions tend to produce *fast* life history strategies, characterized by early maturation, a higher number of sexual partners to whom one is less attached, and less parenting of offspring. Unpredictability and harshness not only affects dispositional social and emotional functioning, but may also promote the development of personality traits linked to higher rates of instability in social relationships or more self-interested behavior. Similarly, detrimental childhood experiences, such as poor parental care or high parent-child conflict, affect personality development and may create a more distrustful, malicious interpersonal style. The aim of this brief review is to survey and summarize findings on the impact of negative early-life experiences on the development of personality and fast life history strategies. By demonstrating that there are parallels in adaptations to adversity in these two domains, we hope to lend weight to current and future attempts to provide a comprehensive insight of personality traits and functions at the ultimate and proximate levels.

There is a superabundance of knowledge about personality, yet research on personality still prospers. The vast majority of such research is, however, aimed at describing certain proximate mechanisms linked to human personality, rather than investigating the possible evolutionary origins or adaptive value of personality traits. Several domains of personality are well-understood, or at least well-examined, for example the structure of personality (e.g., Markon et al., 2005; Bohane et al., 2017) and the effect of early-life experiences on the development of personality traits (e.g., Beaver et al., 2015; Ogle et al., 2015). There are well-established findings about the effect of negative early-life experiences on longitudinal developmental characteristics of personality (e.g., Briley and Tucker-Drob, 2014; Newton-Howes et al., 2015) and on neurobiological processes underlying personality functions (e.g., Kennis et al., 2013; Kundakovic and Champagne, 2015). Similarly, a large number of studies have tested different domains of life history (LH) theory. LH theory is an evolutionary framework explaining and describing the different strategies individuals develop for allocating their limited resources (e.g., energy, time, etc.) adaptively so as to maximize survival and reproduction. Accordingly, LH theory provides predictions about psychosocial processes and their value in terms of survival or reproduction (see Buss, 2015 for an overview). The proximate mechanisms underlying these psychosocial processes include stress responses, different forms of mating and parenting, and adjustment to environmental circumstances (Buss, 2015). Presumably, personality psychology and LH theory, two main domains of different fields of psychology, can contribute to a more comprehensive understanding of psychobiological phenomena, for example by elucidating the long-term effects of early-life stress and the underlying ultimate and proximate mechanisms. In this review, we provide a brief summary of the evidence for the correspondence between reactions to early-stress found in personality and LH research.

Personality characteristics are, to some extent, formed by early-life experiences in the same manner as LH strategies, which alter according to the individual's circumstances. Personality features formed by early adversities may enable behaviors or cognitive-emotional functions which enhance the reproduction and/or survival of the individual under less advantageous conditions. In our view, malevolent or self-centered personality features can be considered to be the joint representative of both early-life adversities and a LH strategy. These malevolent personality traits are partway represented by the Dark Triad, a personality construct consisting of three interrelated traits: Machiavellianism, subclinical psychopathy, and subclinical narcissism (Paulhus and Williams, 2002). In line with recent findings, we suggest that the concept of the Dark Triad provides a feasible model for demonstrating how unpredictable and harsh experiences guide personality development to result in features linked to faster LH strategies.

## Life history traits and life history strategies

Scarcity forces individuals to make trade-offs between actual and future reproduction, or between supporting existing offspring and producing another (Kaplan and Gangestad, 2005). The characteristics that determine the outcome of these trade-offs are the central elements of LH theory and are sometimes referred to as LH traits. LH traits specify the timing of development generally and the development of functions associated with survival and reproduction strategies, such as mating behavior, parental investment, and aging (Kaplan and Gangestad, 2005; Ellis et al., 2009; Bjorklund and Ellis, 2014). Resource limitations compromise individuals' ability to maximize all components of their development, survival, and reproduction. Hence, they must prioritize the allocation of resources through a series of trade-offs. LH theory therefore links LH traits to resource expenditure strategies (Chisholm et al., 1993; Chisholm, 1999; Kaplan and Gangestad, 2005).

The nature of the most adaptive allocation strategy varies according to circumstance. For example, the optimal energy allocation strategies for children and adults are different: children have to invest more in their growth and development to achieve maturity, whilst adults have to support their children and spend energy on their own survival. Consequently, there are individual circumstances and features shaping optimal allocation strategies which can alter across the lifespan and hence may account for differences in the timing of life events (e.g., development, reproduction, etc.) (Chisholm et al., 1993; Chisholm, 1999; Kaplan and Gangestad, 2005; Bjorklund and Ellis, 2014). An individual's configuration of LH traits determines his or her overall LH strategy (Ellis et al., 2009). Thus, LH strategies are adapted to local conditions yet, because of the variation in individual characteristics, they are flexible and diverse within the environmental constraints. In other words, LH strategies are condition-dependent in the sense that behavior can be adapted to the conditions and environmental circumstances that the organism encounters.

## The slow-fast continuum

It has been suggested that all LH traits vary on a slow-fast continuum (Del Giudice et al., 2015). More particular fast and slow LH strategies can be considered as coordinated and integrated patterns of metabolic, cognitive, behavioral, and personality traits (for details see Brumbach et al., 2009; Ellis et al., 2009; Figueredo et al., 2013; Del Giudice et al., 2015; de Baca et al., 2016). Slow LH strategies can be characterized by future oriented attitudes, a relatively long-term focus in behavioral strategies; for example, an ability to delay gratification. These strategies also involve higher parental investment (i.e., investment of time and effort in caring for offspring) in a relatively small number of offspring. In contrast, fast LH strategies are characterized by a relatively short-term focus and present-orientated attitude of taking risks or being aggressive in order to maximize immediate rewards and prioritize mating efforts (Griskevicius et al., 2013; Chen and Chang, 2016). Furthermore, the core of faster strategies is early reproductive maturity, frequent mating, and little investment in social relationships or offspring (Belsky et al., 1991; Nettle, 2010). Many species display a variety of slow and fast traits (Kraus et al., 2005; Bielby et al., 2007). Humans are toward the slow end of the continuum, but also display an array of fast LH traits (Heylighen and Bernheim, 2004; Hawkes, 2006; Ellis et al., 2009).

There are several critical environmental determinants of individuals' slow vs. fast LH strategy trade-offs. The most significant factors are unpredictability, harshness, and resource scarcity (Brumbach et al., 2009; Ellis et al., 2009; Griskevicius et al., 2011; Chang and Lu, 2018). Unpredictability is the predictability of change in the environment, whereas harshness is represented by morbidity-mortality rates. However, there is a lack of correspondence in defining the proximate measures of unpredictability and harshness. Nevertheless, in general harshness is described as the indicator of the extent to which environmental factors cause disability or death at each developmental stage within a population, while unpredictability can be thought of as the spatial and/or temporal variability in harshness (Brumbach et al., 2009; Ellis et al., 2009). Finally, resource scarcity describes the availability of resources and the level of resource competition (Ellis et al., 2009). At higher rates of morbidity-mortality and at a higher variability of conditions (i.e., higher levels of unpredictability and harshness) the risk of dying before producing any offspring is high, thus selection favors faster LH strategies, prompting individuals to start mating relatively early. In contrast, harsh but predictable environments favor slower LH strategies that enhance investments in somatic effort and delay reproduction in order to increase the individual's likelihood of survival and maturation (Ellis et al., 2009; Figueredo et al., 2013; Del Giudice et al., 2015). More generally, early-life environmental indicators of high levels of unpredictability and harshness together with sparse resources favor faster LH strategies involving acceleration of physiological development and sexual maturation (Wilson and Daly, 1997; Ellis, 2004; Belsky et al., 2010). Faster strategies are more beneficial when the future is uncertain and lifespan is unpredictable or mortality and morbidity rates appear to be high (Bereczkei and Csanaky, 2001). Under these conditions, seeking immediate rewards may be adaptive due to the improvement of cost-benefit ratio of resource allocation by reducing cost-ineffective effort toward future fitness payoffs which may not evoke sufficient future benefits (Chen and Chang, 2016). However, less harsh or more predictable circumstances, or access to a suitable amount of resources can alter LH strategies toward a slower form, highlighting the importance of the evaluation process, and how these environmental cues are monitored and interpreted. Environmental conditions during childhood unequivocally form information processing, thus circumstantial factors in early-life shape how the individual reacts to unpredictability and harshness in later-life stages.

## Negative early-life experiences and fast life history strategies

Early-life experiences not only influence personal LH strategy; they may also sensitize individuals to adversity, influencing how they respond to adversity in later life. In other words, adults' reactions to environmental stressors such as harsh or unpredictable environments vary according to their childhood experience of adversity (Griskevicius et al., 2013; White et al., 2013; Chang and Lu, 2018). Earlier research has shown that childhood socioeconomic status (SES) is a reliable indicator of early-life environmental harshness (Griskevicius et al., 2011; Belsky et al., 2012). Low SES is associated with more household stressors (e.g., multiple job changes of parents; unstable or uncertain employment, etc.) which represent greater environmental harshness. Correspondingly, low SES was found to be associated with traits linked to fast LH strategies, such as impulsivity, risk-taking, and unrestricted sociosexual orientation (Brumbach et al., 2009; Belsky et al., 2010; Nettle, 2010; Griskevicius et al., 2011; de Baca et al., 2016.). However, according to more recent findings, it is controversial to apply SES to represent harshness: some research has shown low SES as an indicator of resource scarcity whereas other studies have supported SES as a cue for mortality-morbidity threats (see Griskevicius et al., 2011; Belsky et al., 2012; Chang and Lu, 2018 for more details).

Besides SES, individuals are sensitive to environmental cues of unpredictability (Brumbach et al., 2009; Ellis et al., 2009; Chang and Lu, 2018). For example, early puberty, an unambiguous indicator of fast LH strategy, was found to be associated with adverse family functioning (i.e., indicators of environmental unpredictability) such as paternal absence, high family conflict, and lack of parental care (Belsky, 2012; Ellis, 2013; Chang and Lu, 2018). Furthermore, families with these disadvantageous features frequently experience ecological stressors linked to unpredictability and harshness (e.g., low income, housing insecurity, employment insecurity etc.). Several studies have shown that the presence of ecological stressors in early life favors faster LH strategies leading to certain biodemographic features, such as earlier puberty. Moreover, as a result of these stressors, other proximate behavioral indicators of fast LH strategy may occur, such as opportunistic or exploitative behavior, hostile attitude, and poor social skills (Belsky et al., 1991; Brumbach et al., 2009; Gladden et al., 2013; de Baca et al., 2016; Chang and Lu, 2018).

## Early life stressors and personality development

Early experiences shape the maturation of the individual, influencing subsequent accommodation to actual environments, through regulating the development of personality features. Childhood adversity has been shown to lead to personality dysfunctions and disorders because of the negative influence it exerts on interpersonal competences and socio-cognitive and emotional functioning (Bowlby, 1980; Brumbach et al., 2009; Bjorklund, 2015; de Baca et al., 2016; Jonason et al., 2017). Harsh and random environments have an influence. There is a large body of evidence indicating that negative familial experiences, such as parental absence or parent-offspring conflict, affect behavioral, and neuroendocrine responses to stress (for a review see Ellis, 2013; Anacker et al., 2014). Thus, early exposure to an unstable or unpredictable emotional environment has been found to change the reactions to negative experiences. Earlier research has shown that early-life stressors (e.g., parent-offspring conflict) are positively associated with the development of fast LH strategies (Chisholm et al., 2005; McFarlane et al., 2005; Nettle, 2010; Simpson et al., 2011; Young et al., 2017).

In line with the above findings, it has also been suggested that exposure to an unpredictable environment in childhood is associated with a heightened perception of risk, lower levels of executive functioning, and a more hedonistic attitude (Brumbach et al., 2009; Figueredo et al., 2012; de Baca et al., 2016; Hurst and Kavanagh, 2017). Growing up in harsh conditions influences children's socio-emotional development, promoting the development of hostile and antagonistic interpersonal styles, relatively poor social skills, and lower social and emotional intelligence (Brumbach et al., 2009; de Baca et al., 2016; Hurst and Kavanagh, 2017; Jonason et al., 2017).

The personality features associated with these opportunistic, impulsive, and short-term oriented behavioral strategies (e.g., low agreeableness, low conscientiousness, and high neuroticism) are also associated with indicators of fast LH strategies (McFarlane et al., 2005; Young et al., 2017). In addition, impulsivity, certain aspects of extraversion and openness to experience (e.g., sensation seeking or dominance) are associated with fast LH traits such as a high mortality rate, increased instability in romantic relationships, unrestricted sociosexual orientation promiscuity, and various selfish behaviors (e.g., exploitation, manipulation) (for a review see Del Giudice, 2012, 2014). Some earlier studies have found more direct links between LH strategies and personality development (Rushton, 1985; Belsky et al., 1991; Gladden et al., 2009) and in line with these findings recent studies suggest that certain personality traits are associated with fast LH traits, such as low self-control, short-term mating strategy, and selfish interpersonal behavior. These characteristics are common to Machiavellianism, subclinical psychopathy, and subclinical narcissism, also referred to as the Dark Triad (DT) (Paulhus and Williams, 2002). Several socially-aversive characteristics are common to DT traits: callousness, being manipulative (Jones and Paulhus, 2011), diminished self-control (Jonason and Tost, 2010), selfishness, a more present-oriented time perspective (Birkás and Csathó, 2015), the inability to delay gratification (Brumbach et al., 2009; Birkás et al., 2015a), and being exploitative (McDonald et al., 2012). These characteristics have been shown to be associated primarily with a fast LH strategy (Jonason et al., 2010; McDonald et al., 2012).

## The dark triad: translating early-life adversities into a fast life history strategy

In line with the above mentioned findings, to some extent DT traits can be considered as a collection of personality indicators for fast LH strategies (Figueredo et al., 2005; Jonason et al., 2010, 2012; McDonald et al., 2012). Consequently, the Dark Triad represents a certain cluster of proximate mechanisms related to faster LH strategies.

Another large body of research has shown that negative childhood experiences (e.g., poor parent–child relationship, poor family functioning) are associated with DT traits (Jonason et al., 2014; Láng and Birkás, 2014, 2015; Birkás et al., 2015b; Láng and Lénárd, 2015; Láng and Abell, 2018). Furthermore, recent studies have revealed that several characteristics linked to DT traits are also proximate indicators of fast LH strategies (for a review see Furnham et al., 2013; Vize et al., 2016; Muris et al., 2017). For example, several socioeconomic conditions and psychosocial health outcomes representing trade-offs linked to fast LH strategies were found to be associated with the DT (Jonason et al., 2013, 2015, 2016). Moreover, in line with earlier findings on the effect of early stressors on personality development and personality functions (see above), there is some evidence that DT traits are associated with suboptimal coping strategies (Rim, 1992; Ng et al., 2014; Birkás et al., 2016), reliance on immature ego-defense strategies (Richardson and Boag, 2016), reactive affectivity (Noser et al., 2014), and specific endocrinological reactions (Pfattheicher, 2016). These findings provide some support for the notion that DT traits can be considered as a core for fast LH trade-offs on the proximate level. Personality guides cognitive-affective responses, socioemotional reactions, and behavioral adaptations to current environments. Accordingly, prior studies have found that several cues linked to fast LH strategies (e.g., unrestricted sociosexuality, selfishness, impulsivity, etc.) can be linked to characteristics of the DT. Moreover, similar to LH strategies, the development of the DT traits have been shown to be associated with negative early-life events.

## Conclusion and future directions

The findings reviewed in this paper indicate that stressful early-life experiences shape LH strategies together with personality development. In addition, we aimed to show that self-centered or malevolent personality features can be considered to represent the proximate level of fast LH strategies. Simultaneously, these personality characteristics may enable an examination of the effect of childhood adversities on personality development. More generally, we suggest that personality features are not only the result of environmental conditions, experiences, and genetic factors, but they also enforce evolved trade-offs in order to adapt to actual conditions.

There is some correspondence between the development of fast LH strategies and DT traits, and the proximate representation of these strategies—the patterns and determining factors (i.e., unpredictability and harshness) are similar. Furthermore, DT traits are associated with several behavioral, socio-cognitive, and socio-emotional behaviors similar to those associated with fast LH trade-offs, such as seeking immediate gratification, selfish behaviors (exploitation and manipulation), and maladaptive stress responses. The DT is therefore a useful personality model to consider in relation to fast LH strategies. Early adversity does not necessarily result in pathological development or psychopathology, but it does enhance the probability of negative developmental outcomes. Fast LH strategies represent an adaptation to environmental adversity. To improve the understanding of the evolution of the structure of human personality, it is necessary to integrate findings from research on LH theory and research on personality development. Development of a model of personality that incorporates insights from LH theory might therefore open up new avenues for research.

## Author contributions

ÁC: wrote sections of the manuscript; BB: contributed the conception and wrote sections of the manuscript.

### Conflict of interest statement

The authors declare that the research was conducted in the absence of any commercial or financial relationships that could be construed as a potential conflict of interest.
